# Supplementary material for: HIVprotI: an integrated web based platform for prediction and design of HIV proteins inhibitors
Source: J Cheminform. 2018 Mar 9;10:12. doi: 10.1186/s13321-018-0266-y (PMC5845081; doi:10.1186/s13321-018-0266-y)
Supplement: Supplementary file 2 — Additional file 2. Source code of HIVProtI web server. [file 13321_2018_266_MOESM2_ESM.zip › HIVprotI_Source-code/hivprotI/weka-3-6-6/doc/weka/experiment/CrossValidationResultProducer.html]

CrossValidationResultProducer


---


|  |  |  |
| --- | --- | --- |
| **Overview** \| **Package** \| **Class** \| **Tree** \| **Deprecated** \| **Index** \| **Help** \| **Weka's home** | |  |
| **PREV CLASS**   **NEXT CLASS** | **FRAMES**    **NO FRAMES**     **All Classes** |  |
| SUMMARY: NESTED \| FIELD \| CONSTR \| METHOD | DETAIL: FIELD \| CONSTR \| METHOD |  |


---


## weka.experiment Class CrossValidationResultProducer

```
java.lang.Object
  weka.experiment.CrossValidationResultProducer
```

**All Implemented Interfaces:**: java.io.Serializable, AdditionalMeasureProducer, OptionHandler, RevisionHandler, ResultProducer

---

``` public class CrossValidationResultProducer extends java.lang.Object implements ResultProducer, OptionHandler, AdditionalMeasureProducer, RevisionHandler ```

Generates for each run, carries out an n-fold cross-validation, using the set SplitEvaluator to generate some results. If the class attribute is nominal, the dataset is stratified. Results for each fold are generated, so you may wish to use this in addition with an AveragingResultProducer to obtain averages for each run.


Valid options are:

```
 -X <number of folds>
  The number of folds to use for the cross-validation.
  (default 10)
```

```
 -D
 Save raw split evaluator output.
```

```
 -O <file/directory name/path>
  The filename where raw output will be stored.
  If a directory name is specified then then individual
  outputs will be gzipped, otherwise all output will be
  zipped to the named file. Use in conjuction with -D. (default splitEvalutorOut.zip)
```

```
 -W <class name>
  The full class name of a SplitEvaluator.
  eg: weka.experiment.ClassifierSplitEvaluator
```

```
 Options specific to split evaluator weka.experiment.ClassifierSplitEvaluator:
```

```
 -W <class name>
  The full class name of the classifier.
  eg: weka.classifiers.bayes.NaiveBayes
```

```
 -C <index>
  The index of the class for which IR statistics
  are to be output. (default 1)
```

```
 -I <index>
  The index of an attribute to output in the
  results. This attribute should identify an
  instance in order to know which instances are
  in the test set of a cross validation. if 0
  no output (default 0).
```

```
 -P
  Add target and prediction columns to the result
  for each fold.
```

```
 Options specific to classifier weka.classifiers.rules.ZeroR:
```

```
 -D
  If set, classifier is run in debug mode and
  may output additional info to the console
```

All options after -- will be passed to the split evaluator.

**Version:**
:   $Revision: 1.17 $

**Author:**
:   Len Trigg (trigg@cs.waikato.ac.nz)

**See Also:**: Serialized Form

---

| **Field Summary** | |
| --- | --- |
| `static java.lang.String` | `DATASET_FIELD_NAME`             The name of the key field containing the dataset name |
| `static java.lang.String` | `FOLD_FIELD_NAME`             The name of the key field containing the fold number |
| `static java.lang.String` | `RUN_FIELD_NAME`             The name of the key field containing the run number |
| `static java.lang.String` | `TIMESTAMP_FIELD_NAME`             The name of the result field containing the timestamp |


**Constructor Summary**

`CrossValidationResultProducer()`


| **Method Summary** | |
| --- | --- |
| `void` | `doRun(int run)`             Gets the results for a specified run number. |
| `void` | `doRunKeys(int run)`             Gets the keys for a specified run number. |
| `java.util.Enumeration` | `enumerateMeasures()`             Returns an enumeration of any additional measure names that might be in the SplitEvaluator |
| `java.lang.String` | `getCompatibilityState()`             Gets a description of the internal settings of the result producer, sufficient for distinguishing a ResultProducer instance from another with different settings (ignoring those settings set through this interface). |
| `java.lang.String[]` | `getKeyNames()`             Gets the names of each of the columns produced for a single run. |
| `java.lang.Object[]` | `getKeyTypes()`             Gets the data types of each of the columns produced for a single run. |
| `double` | `getMeasure(java.lang.String additionalMeasureName)`             Returns the value of the named measure |
| `int` | `getNumFolds()`             Get the value of NumFolds. |
| `java.lang.String[]` | `getOptions()`             Gets the current settings of the result producer. |
| `java.io.File` | `getOutputFile()`             Get the value of OutputFile. |
| `boolean` | `getRawOutput()`             Get if raw split evaluator output is to be saved |
| `java.lang.String[]` | `getResultNames()`             Gets the names of each of the columns produced for a single run. |
| `java.lang.Object[]` | `getResultTypes()`             Gets the data types of each of the columns produced for a single run. |
| `java.lang.String` | `getRevision()`             Returns the revision string. |
| `SplitEvaluator` | `getSplitEvaluator()`             Get the SplitEvaluator. |
| `static java.lang.Double` | `getTimestamp()`             Gets a Double representing the current date and time. |
| `java.lang.String` | `globalInfo()`             Returns a string describing this result producer |
| `java.util.Enumeration` | `listOptions()`             Returns an enumeration describing the available options.. |
| `static void` | `main(java.lang.String[] args)`             Quick test of timestamp |
| `java.lang.String` | `numFoldsTipText()`             Returns the tip text for this property |
| `java.lang.String` | `outputFileTipText()`             Returns the tip text for this property |
| `void` | `postProcess()`             Perform any postprocessing. |
| `void` | `preProcess()`             Prepare to generate results. |
| `java.lang.String` | `rawOutputTipText()`             Returns the tip text for this property |
| `void` | `setAdditionalMeasures(java.lang.String[] additionalMeasures)`             Set a list of method names for additional measures to look for in SplitEvaluators. |
| `void` | `setInstances(Instances instances)`             Sets the dataset that results will be obtained for. |
| `void` | `setNumFolds(int newNumFolds)`             Set the value of NumFolds. |
| `void` | `setOptions(java.lang.String[] options)`             Parses a given list of options. |
| `void` | `setOutputFile(java.io.File newOutputFile)`             Set the value of OutputFile. |
| `void` | `setRawOutput(boolean d)`             Set to true if raw split evaluator output is to be saved |
| `void` | `setResultListener(ResultListener listener)`             Sets the object to send results of each run to. |
| `void` | `setSplitEvaluator(SplitEvaluator newSplitEvaluator)`             Set the SplitEvaluator. |
| `java.lang.String` | `splitEvaluatorTipText()`             Returns the tip text for this property |
| `java.lang.String` | `toString()`             Gets a text descrption of the result producer. |

**Methods inherited from class java.lang.Object**

`equals, getClass, hashCode, notify, notifyAll, wait, wait, wait`

**Field Detail**

### DATASET\_FIELD\_NAME

```
public static java.lang.String DATASET_FIELD_NAME
```

:   The name of the key field containing the dataset name

---


### RUN\_FIELD\_NAME

```
public static java.lang.String RUN_FIELD_NAME
```

:   The name of the key field containing the run number

---


### FOLD\_FIELD\_NAME

```
public static java.lang.String FOLD_FIELD_NAME
```

:   The name of the key field containing the fold number

---


### TIMESTAMP\_FIELD\_NAME

```
public static java.lang.String TIMESTAMP_FIELD_NAME
```

:   The name of the result field containing the timestamp


**Constructor Detail**

### CrossValidationResultProducer

```
public CrossValidationResultProducer()
```


**Method Detail**

### globalInfo

```
public java.lang.String globalInfo()
```

:   Returns a string describing this result producer

    :   **Returns:**: a description of the result producer suitable for displaying in the explorer/experimenter gui

---


### setInstances

```
public void setInstances(Instances instances)
```

:   Sets the dataset that results will be obtained for.

    :   **Specified by:**: `setInstances` in interface `ResultProducer`
    :   **Parameters:**: `instances` - a value of type 'Instances'.

---


### setResultListener

```
public void setResultListener(ResultListener listener)
```

:   Sets the object to send results of each run to.

    :   **Specified by:**: `setResultListener` in interface `ResultProducer`
    :   **Parameters:**: `listener` - a value of type 'ResultListener'

---


### setAdditionalMeasures

```
public void setAdditionalMeasures(java.lang.String[] additionalMeasures)
```

:   Set a list of method names for additional measures to look for
    in SplitEvaluators. This could contain many measures (of which only a
    subset may be produceable by the current SplitEvaluator) if an experiment
    is the type that iterates over a set of properties.

    :   **Specified by:**: `setAdditionalMeasures` in interface `ResultProducer`
    :   **Parameters:**: `additionalMeasures` - an array of measure names, null if none

---


### enumerateMeasures

```
public java.util.Enumeration enumerateMeasures()
```

:   Returns an enumeration of any additional measure names that might be
    in the SplitEvaluator

    :   **Specified by:**: `enumerateMeasures` in interface `AdditionalMeasureProducer`
    :   **Returns:**: an enumeration of the measure names

---


### getMeasure

```
public double getMeasure(java.lang.String additionalMeasureName)
```

:   Returns the value of the named measure

    :   **Specified by:**: `getMeasure` in interface `AdditionalMeasureProducer`
    :   **Parameters:**: `additionalMeasureName` - the name of the measure to query for its value **Returns:**: the value of the named measure **Throws:**: `java.lang.IllegalArgumentException` - if the named measure is not supported

---


### getTimestamp

```
public static java.lang.Double getTimestamp()
```

:   Gets a Double representing the current date and time.
    eg: 1:46pm on 20/5/1999 -> 19990520.1346

    :   **Returns:**: a value of type Double

---


### preProcess

```
public void preProcess()
                throws java.lang.Exception
```

:   Prepare to generate results.

    :   **Specified by:**: `preProcess` in interface `ResultProducer`
    :   **Throws:**: `java.lang.Exception` - if an error occurs during preprocessing.

---


### postProcess

```
public void postProcess()
                 throws java.lang.Exception
```

:   Perform any postprocessing. When this method is called, it indicates
    that no more requests to generate results for the current experiment
    will be sent.

    :   **Specified by:**: `postProcess` in interface `ResultProducer`
    :   **Throws:**: `java.lang.Exception` - if an error occurs

---


### doRunKeys

```
public void doRunKeys(int run)
               throws java.lang.Exception
```

:   Gets the keys for a specified run number. Different run
    numbers correspond to different randomizations of the data. Keys
    produced should be sent to the current ResultListener

    :   **Specified by:**: `doRunKeys` in interface `ResultProducer`
    :   **Parameters:**: `run` - the run number to get keys for. **Throws:**: `java.lang.Exception` - if a problem occurs while getting the keys

---


### doRun

```
public void doRun(int run)
           throws java.lang.Exception
```

:   Gets the results for a specified run number. Different run
    numbers correspond to different randomizations of the data. Results
    produced should be sent to the current ResultListener

    :   **Specified by:**: `doRun` in interface `ResultProducer`
    :   **Parameters:**: `run` - the run number to get results for. **Throws:**: `java.lang.Exception` - if a problem occurs while getting the results

---


### getKeyNames

```
public java.lang.String[] getKeyNames()
```

:   Gets the names of each of the columns produced for a single run.
    This method should really be static.

    :   **Specified by:**: `getKeyNames` in interface `ResultProducer`
    :   **Returns:**: an array containing the name of each column

---


### getKeyTypes

```
public java.lang.Object[] getKeyTypes()
```

:   Gets the data types of each of the columns produced for a single run.
    This method should really be static.

    :   **Specified by:**: `getKeyTypes` in interface `ResultProducer`
    :   **Returns:**: an array containing objects of the type of each column. The objects should be Strings, or Doubles.

---


### getResultNames

```
public java.lang.String[] getResultNames()
```

:   Gets the names of each of the columns produced for a single run.
    This method should really be static.

    :   **Specified by:**: `getResultNames` in interface `ResultProducer`
    :   **Returns:**: an array containing the name of each column

---


### getResultTypes

```
public java.lang.Object[] getResultTypes()
```

:   Gets the data types of each of the columns produced for a single run.
    This method should really be static.

    :   **Specified by:**: `getResultTypes` in interface `ResultProducer`
    :   **Returns:**: an array containing objects of the type of each column. The objects should be Strings, or Doubles.

---


### getCompatibilityState

```
public java.lang.String getCompatibilityState()
```

:   Gets a description of the internal settings of the result
    producer, sufficient for distinguishing a ResultProducer
    instance from another with different settings (ignoring
    those settings set through this interface). For example,
    a cross-validation ResultProducer may have a setting for the
    number of folds. For a given state, the results produced should
    be compatible. Typically if a ResultProducer is an OptionHandler,
    this string will represent the command line arguments required
    to set the ResultProducer to that state.

    :   **Specified by:**: `getCompatibilityState` in interface `ResultProducer`
    :   **Returns:**: the description of the ResultProducer state, or null if no state is defined

---


### outputFileTipText

```
public java.lang.String outputFileTipText()
```

:   Returns the tip text for this property

    :   **Returns:**: tip text for this property suitable for displaying in the explorer/experimenter gui

---


### getOutputFile

```
public java.io.File getOutputFile()
```

:   Get the value of OutputFile.

    :   **Returns:**: Value of OutputFile.

---


### setOutputFile

```
public void setOutputFile(java.io.File newOutputFile)
```

:   Set the value of OutputFile.

    :   **Parameters:**: `newOutputFile` - Value to assign to OutputFile.

---


### numFoldsTipText

```
public java.lang.String numFoldsTipText()
```

:   Returns the tip text for this property

    :   **Returns:**: tip text for this property suitable for displaying in the explorer/experimenter gui

---


### getNumFolds

```
public int getNumFolds()
```

:   Get the value of NumFolds.

    :   **Returns:**: Value of NumFolds.

---


### setNumFolds

```
public void setNumFolds(int newNumFolds)
```

:   Set the value of NumFolds.

    :   **Parameters:**: `newNumFolds` - Value to assign to NumFolds.

---


### rawOutputTipText

```
public java.lang.String rawOutputTipText()
```

:   Returns the tip text for this property

    :   **Returns:**: tip text for this property suitable for displaying in the explorer/experimenter gui

---


### getRawOutput

```
public boolean getRawOutput()
```

:   Get if raw split evaluator output is to be saved

    :   **Returns:**: true if raw split evalutor output is to be saved

---


### setRawOutput

```
public void setRawOutput(boolean d)
```

:   Set to true if raw split evaluator output is to be saved

    :   **Parameters:**: `d` - true if output is to be saved

---


### splitEvaluatorTipText

```
public java.lang.String splitEvaluatorTipText()
```

:   Returns the tip text for this property

    :   **Returns:**: tip text for this property suitable for displaying in the explorer/experimenter gui

---


### getSplitEvaluator

```
public SplitEvaluator getSplitEvaluator()
```

:   Get the SplitEvaluator.

    :   **Returns:**: the SplitEvaluator.

---


### setSplitEvaluator

```
public void setSplitEvaluator(SplitEvaluator newSplitEvaluator)
```

:   Set the SplitEvaluator.

    :   **Parameters:**: `newSplitEvaluator` - new SplitEvaluator to use.

---


### listOptions

```
public java.util.Enumeration listOptions()
```

:   Returns an enumeration describing the available options..

    :   **Specified by:**: `listOptions` in interface `OptionHandler`
    :   **Returns:**: an enumeration of all the available options.

---


### setOptions

```
public void setOptions(java.lang.String[] options)
                throws java.lang.Exception
```

:   Parses a given list of options.


    Valid options are:

    ```
     -X <number of folds>
      The number of folds to use for the cross-validation.
      (default 10)
    ```

    ```
     -D
     Save raw split evaluator output.
    ```

    ```
     -O <file/directory name/path>
      The filename where raw output will be stored.
      If a directory name is specified then then individual
      outputs will be gzipped, otherwise all output will be
      zipped to the named file. Use in conjuction with -D. (default splitEvalutorOut.zip)
    ```

    ```
     -W <class name>
      The full class name of a SplitEvaluator.
      eg: weka.experiment.ClassifierSplitEvaluator
    ```

    ```
     Options specific to split evaluator weka.experiment.ClassifierSplitEvaluator:
    ```

    ```
     -W <class name>
      The full class name of the classifier.
      eg: weka.classifiers.bayes.NaiveBayes
    ```

    ```
     -C <index>
      The index of the class for which IR statistics
      are to be output. (default 1)
    ```

    ```
     -I <index>
      The index of an attribute to output in the
      results. This attribute should identify an
      instance in order to know which instances are
      in the test set of a cross validation. if 0
      no output (default 0).
    ```

    ```
     -P
      Add target and prediction columns to the result
      for each fold.
    ```

    ```
     Options specific to classifier weka.classifiers.rules.ZeroR:
    ```

    ```
     -D
      If set, classifier is run in debug mode and
      may output additional info to the console
    ```

    All options after -- will be passed to the split evaluator.

    :   **Specified by:**: `setOptions` in interface `OptionHandler`
    :   **Parameters:**: `options` - the list of options as an array of strings **Throws:**: `java.lang.Exception` - if an option is not supported

---


### getOptions

```
public java.lang.String[] getOptions()
```

:   Gets the current settings of the result producer.

    :   **Specified by:**: `getOptions` in interface `OptionHandler`
    :   **Returns:**: an array of strings suitable for passing to setOptions

---


### toString

```
public java.lang.String toString()
```

:   Gets a text descrption of the result producer.

    :   **Overrides:**: `toString` in class `java.lang.Object`
    :   **Returns:**: a text description of the result producer.

---


### getRevision

```
public java.lang.String getRevision()
```

:   Returns the revision string.

    :   **Specified by:**: `getRevision` in interface `RevisionHandler`
    :   **Returns:**: the revision

---


### main

```
public static void main(java.lang.String[] args)
```

:   Quick test of timestamp

    :   **Parameters:**: `args` - the commandline options


---


|  |  |  |
| --- | --- | --- |
| **Overview** \| **Package** \| **Class** \| **Tree** \| **Deprecated** \| **Index** \| **Help** \| **Weka's home** | |  |
| **PREV CLASS**   **NEXT CLASS** | **FRAMES**    **NO FRAMES**     **All Classes** |  |
| SUMMARY: NESTED \| FIELD \| CONSTR \| METHOD | DETAIL: FIELD \| CONSTR \| METHOD |  |


---
